# Evaluation of cardiac function in asthmatic children by Tissue Doppler Echocardiography

**DOI:** 10.1186/s43044-023-00363-4

**Published:** 2023-05-03

**Authors:** Reham Wagdy, Ghada El-Deriny

**Affiliations:** Present Address: Department of Pediatrics, Faculty of Medicne, Alexandria University, Alexandria, Egypt

**Keywords:** Tricuspid annular plane systolic excursion, Peak expiratory flow rate, Isovolumic relaxation time, Pulmonary function test, Ventricular dysfunction

## Abstract

**Background:**

Bronchial asthma is a global health problem with rising prevalence in developing countries. Children with severe asthma can experience cor pulmonale later in life, but little is known about the cardiac changes that might be present earlier in mild or moderate severity of disease. This study aimed to evaluate biventricular function among children suffering from persistent asthma by Tissue Doppler Echocardiography (TDE).

**Results:**

Thirty-five asthmatic children enrolled from Alexandria Children’s Hospital between September 2021 to May 2022; they were compared to 35 healthy matched children. Chronic respiratory disease, cardiac disease, or other comorbidities were excluded. The mean age of cases was 8.87 ± 2.03 years with a male/female ratio of 54.3%:45.7%. There were 28.3% mild cases, 45.7% moderate, and 25.7% severe. Conventional echocardiographic variables of cardiac function were normal for both ventricles. The TDE indices of medial mitral annulus (*S*’ velocity and peak *E*’) were significantly reduced (14.55 ± 2.30 and 14.69 ± 2.30) versus controls (15.68 ± 1.96, 15.69 ± 1.76, *P*; 0.044, *P* < 0.0045) but with preserved LV function. The lateral tricuspid annulus (*S*’ velocity and peak *E*’) was significantly reduced (11.53 ± 3.24 and 11.56 ± 3.18) versus controls (15.71 ± 0.98, 16.02 ± 1.75, < 0.001*), while *E*/*A* and IVRT were significantly increased (1.49 ± 0.06 versus 1.70 ± 0.18 and 102.39 ± 5.37 versus 140.10 ± 34.35, respectively, *P* < 0.001*) with impaired RV function. Peak expiratory flow rate (PEFR) was negatively correlated with the IVRT of the tricuspid annulus (*P* = 0.002, *r* = −0.503*) and to the *E*'/*A*' (*P* = 0.036, *r* =−0.355*). All TDE variables of lateral tricuspid annulus of severe subgroups were significantly  changed versus moderate or mild subgroups.

**Conclusions:**

Tissue Doppler echocardiography is the recommended modality for early detection of biventricular cardiac dysfunction among children with different levels of asthma severity. Periodic screening is advised through the use of IVRT especially for RV.

## Background

Bronchial asthma is a common respiratory disorder among children, worldwide. Asthma is characterized by chronic inflammation and remodeling of the airways induced by recurrent exposure to hypoxemia that leads to repeated tissue injury and repair. The interaction between respiratory diseases and cardiovascular function is complex [1, 2].

Cardiac dysfunction can be attributed to pulmonary hypertension (PH) secondary to recurrent hypoxia in patients with bronchial asthma. PH affects the pulmonary vasculature by releasing various cytokines leading to pulmonary vasoconstriction and enhancing the remodeling process with muscularization and proliferation of the vascular media and intima [3–5]. Other hypotheses concluded that the exaggerated respiratory efforts may raise the intrathoracic pressure and increase right ventricle (RV) afterload and consequently RV hypertrophy and/or dilatation [6].

Children with severe bronchial asthma can experience cor pulmonale later in life, but little is known about the early cardiac changes that might be present during childhood especially for mild or moderate persistent asthma. Some studies have reported right ventricular dysfunction as the earliest hemodynamic change among those cases [7, 8]. Other studies did not report these results and reported impaired systolic function of the left ventricle (LV) even before diastolic dysfunction [9]. This controversy is multifactorial. It could be attributed to the absence or presence of PH, degree of severity of persistent asthma, and its impact on cardiac performance or even interaction between the RV and LV related performance. An important factor that could also be the cause is the difficult assessment of right ventricular function by conventional echocardiography because of its position back to the sternum or to its irregular shape and geometry [10]. However, Tissue Doppler Echocardiography (TDE) had conquered this problem and offers an accurate assessment of the global and regional function of both ventricles [8]. This work was conducted to evaluate biventricular cardiac function in children suffering from persistent asthma by Tissue Doppler Echocardiography (TDE) versus conventional echocardiography.

## Methods

### Study design and settings

This was a case–control study conducted at the Main Children’s Hospital from September 2021 to May 2022. Ethical approval was received from the ethical and research committee of Faculty of Medicine No: 0305278. Informed consent was taken from the caregivers of all participants.

#### Sample size

We calculated the sample size using G Power 3.1.9.7, 2020. Based on a mean TDE mitral E′ velocity among asthmatic patients of 0.17 ± 0.03 and among controls of 0.19 ± 0.03, and a mean TDE tricuspid S′ velocity among asthmatic patients of 0.08 ± 0.022 and among controls of 0.10 ± 0.028 (1), alpha error of 0.05, power of 80%, a ratio of cases to controls of 1:1, the minimum required sample size was calculated to be 68 (34 asthmatic patients and 34 controls). The sample size was calculated based on a previous study [9].

### Study population

The study was conducted over two groups of children.

- ***Group I (patients):*** This group included 35 asthmatic children diagnosed according to updated GINA guidelines 2019 with different levels of asthma severity whether controlled or not controlled on medications [11]. The cases were selected consecutively from pulmonology clinic of Alexandria Main Children’s Hospital during follow-up visits.
- **Inclusion criteria:** The age of the enrolled cases ranged between 5 and 15 years (to avoid transient wheezes). The duration of the disease was at least one year. The cases had been on inhalation steroids for at least six months, and the last exacerbation was at least one month. The minimum oxygen saturation was 91%, and the hemoglobin level was 11 g/dl.
- **Exclusion criteria:** We excluded patients with acute asthma exacerbation, patients with chronic respiratory comorbidities, e.g., interstitial lung disease, congenital lung anomalies, patients with clinical evidence of right ventricular failure, patients with other comorbid diseases, positive cardiac history of congenital heart disease or acquired heart disease, gastroesophageal reflux, obesity, sleep apnea, and/or anemia or polycythemia.
- ***Group II (control):*** Thirty-five healthy children of matched age and gender were included as controls.

### Investigations

All participants had a history and physical examination. Group I was further subdivided according to degree of asthma severity: mild, moderate, and severe. Transthoracic echocardiography and pulmonary function tests were also done [11, 12].

1. Pulmonary function tests
  Peak expiratory flow rate was evaluated using a mini-Wright peak flow meter. The best reading from three forced expirations was recorded.
  Spirometry was measured via forced expiratory volume in 1 s (FEV1).

Pulmonary function testing (PFT) was performed using CareFusion Germany 234 GmbH pulmonary function apparatus according to new American Thoracic Society (ATS) and European Respiratory Society (ERS) standardization guidelines for performance of spirometry [12].

2. Echocardiography study [13]

Transthoracic echocardiography was performed by a single experienced pediatric cardiologist blinded from the respiratory status of the case at the cardiology clinic of the Main Children University Hospital. The studies were performed for cases and controls using a Philips machine HD 11, 2–5 MHz two-phase array imaging transducer. These studies aimed to assess parameters of systolic and diastolic function of both ventricles based on the recommendations of the American Society of Echocardiography. The left and right ventricular functions were assessed via two-dimensional echocardiography: M-mode, color-flow imaging, standard pulsed-wave Doppler and TDE according to Guidelines of the American Society of Echocardiography [13].

- **Conventional echocardiography**

Conventional echocardiography (M-mode, 2-D echo, and Doppler study) measured the following parameters: ejection fraction (EF, %), fractional shortening (FS, %), tricuspid annular plane systolic excursion (TAPSE), E velocity (cm/sec; peak velocity during early diastole), A velocity (cm/sec; peak velocity during late diastole), and the E/A ratio of mitral and tricuspid valves. Pulmonary artery pressure was estimated (if possible) through calculations of the Bernoulli equation after measuring tricuspid and pulmonary regurgitation and estimating the pressure gradient. Normal RAP was considered 5 mmHg. PSAP = tricuspid regurgitation gradient + RAP. PSAP = (*V*_max_^2^ × 4) + RAP. Normal systolic arterial pressure is up to 30 mmHg at rest [13].

- **Tissue Doppler Echocardiography (TDE)**

The TDE modality was performed in apical four-chamber planes with the pulsed-wave Doppler sample volume placed successively at the tip of the lateral tricuspid annulus midway between the apex of the right ventricle and the tricuspid annulus. Similarly, the sample volume was placed at the tip of the lateral and medial mitral annulus at an apical four-chamber view.

The TDE was used to assess S' velocity (cm/sec; peak systolic velocity), E' velocity (cm/sec; peak early diastolic velocity), A' velocity (cm/sec; peak late diastolic velocity), the E’/A’ ratio, and isovolumetric relaxation time IVRT, from the end of the S-wave to the beginning of E-wave. These were measured at lateral leaflets of mitral and tricuspid annuli and at the medial leaflets of the mitral annulus. At least three clearest successive cycles were used for calculations. These steps were done in addition to estimating medial and lateral mitral annuli *E*/*E*’ as an indicator of left ventricular filling pressure [13].

### Statistical analysis of the data

Data were fed to the computer and analyzed using IBM SPSS software package version 20.0 (Armonk, NY: IBM Corp). Qualitative data were described using number and percent. The Kolmogorov–Smirnov test was used to verify the normality of distribution quantitative data described using range (minimum and maximum), mean, standard deviation, and median. The significance of the obtained results was judged at the 5% level. Student’s t test was used for normally distributed quantitative variables and to compare between two studied groups. An F-test (ANOVA) was used for normally distributed quantitative variables and to compare between more than two groups. A post hoc test (Tukey’s test) was used for pairwise comparisons. Pearson’s coefficient was used to correlate between two normally distributed quantitative variables.

## Results

We enrolled 35 asthmatic children: 19 males (54.3%) and 16 females (45.7%) with a mean age of 8.87 ± 2.03 years. The characteristics of the asthmatic groups and controls were similar in terms of age, gender, weight, height, and body mass index (BMI) as shown in Table 1. Pulmonary function tests including PERF and FEV1 were significantly lower in the asthmatic group than the control group (Table 1). All asthmatic patients were clinically stable. Of the 35 patients, 28.3% cases presented mild asthma, 45.71% moderate asthma, and 25.7% suffered from very severe bronchial asthma. The mean PEFR of severe asthmatic (159.6 ± 35.9) was significantly lower than moderate and mild disease (200.6 ± 35.1 and 238.3 ± 36.6, respectively, *P* < 0.001*). There was no statistically significant difference between mild asthmatics and the control group (224.7 ± 42.7).

**Table 1:** Demographic data, anthropometric data, and peak expiratory flow rate among the studied asthmatic children and controls

Data	Group II “Control” (*n* = 35)	Group I “Asthma” (*n* = 35)	Test of significance
Age (years)			
Min.–Max	5.0–13.0	5.0–13.0	^t^*p* = 1.00
Mean ± SD	8.87 ± 2.33	8.87 ± 2.03	
Sex			
Male	19 (54.3%)	19 (54.3)	χ^2^*p* = 1.000
Female	16 (45.7)	16 (45.7)	
Weight (kg)			
Min.–Max	18.0–50.0	16.0–58.0	0.295
Mean ± SD	32.21 ± 8.60	29.93 ± 9.51	
Height (cm)			
Min.–Max	115.0–151.0	105.0–152.0	0.365
Mean ± SD	133.43 ± 10.0	131.17 ± 10.69	
BMI (kg/m^2^)			
Min.–Max	12.90–25.0	12.42–28.90	0.250
Mean ± SD	17.87 ± 2.78	17.0 ± 3.48	
Heart rate			
Min.–Max	84.0–112.0	85.0–110.0	0.676
Mean ± SD	95.86 ± 6.97	95.17 ± 6.68	
FEV1			
Min.–Max	82.0–100.0	38.0–93.0	8.451*
Mean ± SD	95.3 ± 18.3	69.26 ± 15.31	< 0.001*
PEFR (L/sec)			
Min.–Max	150.0–300.0	100.0–280.0	0.027*
Mean ± SD	224.71 ± 42.74	200.83 ± 45.46	

*Statistically significant at* p* ≤ 0.05

## Echocardiographic characteristics

Conventional echocardiographic variables of LV and RV  such as EF, FS, TAPSE, E velocity, A velocity, and E/A did not differ significantly between patients and controls (*P* > 0.05) as shown in Tables 2 and 6. Pulmonary hypertension was not reported among the asthmatic group.

**Table 2:** Comparison of the LV function of asthmatic children and controls by conventional echocardiography and TDE

Left ventricular function	Group I “Control” (*n* = 35)		Group II “Asthma” (*n* = 35)		*p*
	Min.–Max	Mean ± SD	Min.–Max	Mean ± SD	
Conventional echocardiography					
EF %	56.0–78.0	66.43 ± 5.98	60.0–75.0	64.97 ± 11.98	0.522
FS %	28.0–44.0	34.46 ± 4.03	27.0–44.0	33.54 ± 4.25	0.359
*E* velocity (cm/sec)	78.0–105.0	91.41 ± 7.2	84.0–105.0	93.64 ± 5.64	0.154
*A* velocity (cm/sec)	38.0–65.0	53.94 ± 7.30	38.70–62.0	50.56 ± 5.98	0.329
*E*/*A* ratio	1.30–2.0	1.71 ± 0.18	1.44–2.39	1.79 ± 0.21	0.093
Pulsed-wave Tissue Doppler					
*Medial leaflets of mitral annulus*					
*S*' velocity(cm/sec)	11.80–18.10	15.68 ± 1.96	9.70–17.20	14.55 ± 2.30	0.044*
*E*' velocity (cm/sec)	10.90–18.0	15.69 ± 1.76	9.60–17.60	14.69 ± 2.30	0.045*
*A*' velocity(cm/sec)	8.10–15.20	11.51 ± 1.80	1.30–14.30	10.65 ± 2.59	0.109
*E*'/*A*' ratio	1.10–1.70	1.36 ± 0.17	1.10–1.80	1.36 ± 0.18	0.920
IVRT msec	54.90–69.0	62.15 ± 3.58	56.80–79.80	68.50 ± 5.39	< 0.001*
*E*/*E*'	4.62–8.84	5.92 ± 1.0	4.94–9.90	6.56 ± 1.32	< 0.025*
*Lateral leaflets of mitral annulus*					
*S*' velocity(cm/sec)	10.90–18.10	15.04 ± 1.94	10.20–18.0	14.76 ± 2.41	0.591
*E*' velocity (cm/sec)	11.30–18.20	15.11 ± 1.91	11.20–19.0	14.84 ± 2.03	0.574
*A*' velocity(cm/sec)	8.90–14.10	11.26 ± 1.51	7.80–14.40	10.79 ± 1.56	0.203
*E*'/*A*' ratio	1.04–1.60	1.32 ± 0.13	1.10–1.90	1.36 ± 0.19	0.228
IVRT msec	51.90–70.0	59.67 ± 3.68	58.80–75.0	65.99 ± 5.40	< 0.001*
*E*/*E*'	4.41–8.50	6.16 ± 0.99	4.94–9.03	6.43 ± 0.98	< 0.258

Mitral value peak *E* velocity (cm/sec), Mitral value peak *A* velocity (cm/sec), Mitral value *E*/*A* ratio

*p*: *p* value for comparing between the studied groups

*Statistically significant at *p* ≤ 0.05

**Fig. 1:**
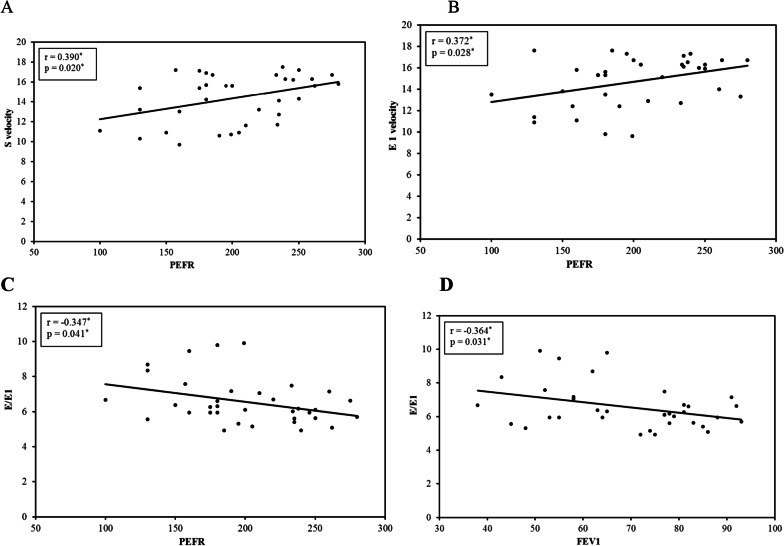
**A–D** Correlation of TDE findings at medial mitral annulus with pulmonary function test among asthmatic group. **A** Correlation of PEFR with S' velocity at medial mitral annulus among asthmatic group. **B** Correlation of PEFR with E' velocity at medial mitral annulus among asthmatic group. **C** Correlation of PEFR with E/E' at medial mitral annulus among asthmatic group. **D** Correlation of FEV1with *E*/*E*' at medial mitral annulus among asthmatic group

Concerning the TDE study of LV at mitral annulus, the mean of the *S*' velocity at the medial mitral annulus was significantly lower in asthmatic children than controls (14.55 ± 2.30 vs. 15.68 ± 1.96; *P* = 0.044). Similarly, the *E*' velocity of the medial mitral annulus (14.69 ± 2.30) was significantly lower for asthmatics than the control group (15.69 ± 1.76; *P* < 0.0045). The *E*’/*A*’ ratios at the medial mitral annulus were significantly greater in patients than the control group. The IVRT was significantly (*P* < 0.001) greater in the asthmatic group at both medial and lateral leaflets of mitral annulus (68.50 ± 5.39 ms and 65.99 ± 5.40, respectively) in relation to control group (62.15 ± 3.58 ms and 59.67 ± 3.68, respectively) as summarized in Table 2. In contrast, the *A*' velocity and *E*'/*A*' ratio did not differ significantly among the studied groups at lateral mitral annulus. There was a positive correlation between S' and E' velocities of medial mitral annulus and PFT (PEFR and FEV1). Negative correlation was found between *E*/*E*' and PFT (PEFR, FEV1**)** (*P* < 0.001) as shown in Fig. 1A–D.

Further comparative study of TDE variables of LV among asthmatic subgroups with mild, moderate, and severe disease is shown in Tables 3 and 4. The severe asthmatic subgroup had significantly lower values of *S*' and *E*' velocities at medial mitral annulus and higher E/E' ratios versus controls as well as between mild and moderate disease (Table 3). However, the findings of these parameters did not differ significantly between mild or moderate asthmatic patients and the control group. The IVRT at medial mitral annulus was significantly greater (*P* < 0.001) for all subgroups even in the mild group versus controls. IVRT was the only parameter of lateral mitral annulus significantly greater among the moderate and severe subgroups when compared to the control group (Table 4).

**Table 3:** Comparison between the asthmatic subgroups and controls according to TDE variables at medial mitral annulus

Medial mitral annulus	Mild (*n* = 10)	Moderate (*n* = 16)	Severe (*n* = 9)	Control (*n* = 35)	*F*	*p*
*S′* velocity (cm/sec)						
Min.–Max	13.20–16.90	10.90–17.50	9.70–17.20	11.80–18.10	6.245*	0.001*
Mean ± SD	15.45 ± 1.22	14.86 ± 2.28	12.18 ± 2.55	15.68 ± 1.96		
Median	15.70	15.65	11.10	15.90		
p_1_	1.000	0.827	< 0.001*			
Sig.bet.Grps	*p*_2_ = 0.888, *p*_3_ = 0.005*, *p*_4_ = 0.013*					
*E′ * velocity (cm/sec)						
Min.–Max	13.30–16.70	9.80–17.60	9.60–17.60	10.90–18.0	5.046*	0.003*
Mean ± SD	15.51 ± 1.13	15.18 ± 2.31	12.91 ± 2.49	15.69 ± 1.76		
Median	15.80	16.10	12.40	16.10		
p_1_	0.994	0.815	0.002*			
Sig.bet.Grps	*p*_2_ = 0.973, *p*_3_ = 0.024*, *p*_4_ = 0.032*					
*E*/*E′ *						
Min.–Max	5.09–7.14	4.94–9.80	5.57–9.90	4.62–8.84	5.026*	0.003*
Mean ± SD	6.11 ± 0.66	6.31 ± 1.32	7.52 ± 1.48	5.92 ± 1.0		
Median	6.11	6.06	7.18	5.81		
*p*_1_	0.965	0.662	0.001*			
Sig.bet.Grps	*p*_2_ = 0.971, *p*_3_ = 0.036*, *p*_4_ = 0.051					
*A′* velocity (cm/sec)						
Min.–Max	9.90–13.70	6.10–14.30	5.60–11.10	8.10–15.20	3.149	0.031*
Mean ± SD	11.43 ± 1.37	11.39 ± 2.24	9.46 ± 1.60	11.51 ± 1.80		
Median	11.30	11.40	9.70	11.10		
*p*_1_	0.999	0.996	0.020*			
Sig.bet.Grps	*p*_2_ = 1.000, *p*_3_ = 0.099, *p*_4_ = 0.065					
*E′*/*A′* ratio						
Min.–Max	1.20–1.68	1.10–1.80	1.16–1.70	1.10–1.70	0.353	0.787
Mean ± SD	1.39 ± 0.15	1.32 ± 0.18	1.37 ± 0.22	1.36 ± 0.17		
Median	1.41	1.28	1.26	1.37		
IVRT msec						
Min.–Max	56.80–71.40	63.90–79.0	58.80–79.80	54.90–69.0	12.298*	< 0.001*
Mean ± SD	66.61 ± 5.31	69.67 ± 4.21	68.52 ± 7.15	62.15 ± 3.58		
Median	69.75	68.80	69.20	62.40		
*p*_1_	0.039*	< 0.001*	0.002*			
Sig.bet.Grps	*p*_2_ = 0.348, *p*_3_ = 0.797, *p*_4_ = 0.930					

*F*: *F* for ANOVA test; pairwise comparison bet. each of the 2 groups was done using post hoc test (Tukey)

*p*: *p* value for comparing between the studied groups

*p*_1_: *p* value for comparing between control and each other group

*p*_2_: *p* value for comparing between mild and moderate

*p*_3_: *p* value for comparing between mild and severe

*p*_4_: *p* value for comparing between moderate and severe

*Statistically significant at *p* ≤ 0.05

**Table 4:** Comparison between the asthmatic subgroups and controls according to TDE variables at lateral Mitral Annulus

Lateral mitral annulus	Mild (*n* = 10)	Moderate (*n* = 16)	Severe (*n* = 9)	Control (*n* = 35)	*F*	*p*
*S′* velocity (cm/sec)						
Min.–Max	10.50–17.10	10.70–18.0	10.20–16.90	10.90–18.10	0.857	0.468
Mean ± SD	14.37 ± 2.01	15.36 ± 2.46	14.12 ± 2.73	15.04 ± 1.94		
Median	14.15	16.30	15.20	15.30		
*E′ * velocity (cm/sec)						
Min.–Max	11.80–18.0	11.30–17.30	11.20–19.0	11.30–18.20	0.596	0.620
Mean ± SD	14.88 ± 1.76	14.47 ± 1.87	15.47 ± 2.58	15.11 ± 1.91		
Median	15.10	14.35	16.20	15.80		
*E*/*E′ *						
Min.–Max	5.28–8.05	4.94–9.03	5.16–8.39	4.41–8.50	0.601	0.617
Mean ± SD	6.40 ± 0.75	6.54 ± 1.04	6.25 ± 1.14	6.16 ± 0.99		
Median	6.44	6.70	5.94	6.08		
A′ velocity (cm/sec)						
Min.–Max	9.30–14.40	7.80–13.20	9.20–12.30	8.90–14.10	2.718	0.052
Mean ± SD	11.74 ± 1.59	10.23 ± 1.56	10.74 ± 1.05	11.26 ± 1.51		
Median	11.90	9.70	11.10	11.20		
*E′*/*A′* ratio						
Min.–Max	1.10–1.50	1.10–1.90	1.20–1.60	1.04–1.60	2.296	0.086
Mean ± SD	1.27 ± 0.14	1.41 ± 0.23	1.40 ± 0.13	1.32 ± 0.13		
Median	1.24	1.40	1.40	1.30		
IVRT msec						
Min.–Max	56.80–71.60	61.70–75.0	58.90–72.90	51.90–70.0	15.575*	< 0.001*
Mean ± SD	62.66 ± 5.64	68.24 ± 4.36	65.67 ± 5.34	59.67 ± 3.68		
Median	60.70	68.45	65.90	60.20		
*p*_1_	0.233	< 0.001*	0.003*			
Sig.bet.Grps	*p*_2_ = 0.012*, *p*_3_ = 0.445, *p*_4_ = 0.494					

*F*: *F* for ANOVA test; pairwise comparison bet. each of the 2 groups was done using post hoc test (Tukey)

*p*: *p* value for comparing between the studied groups

*p*_1_: *p* value for comparing between control and each other group

*p*_2_: *p* value for comparing between mild and moderate

*p*_3_: *p* value for comparing between mild and severe

*p*_4_: *p* value for comparing between moderate and severe

*Statistically significant at *p* ≤ 0.05

Tissue Doppler echocardiography variables of RV at the lateral tricuspid annulus showed that all the studied parameters were significantly different among the asthmatic group (*P* < 0.01) versus controls (Table 5). Analyzing TD data of the asthmatic subgroups explained this significant difference. The S' velocities of mild, moderate, and severe subgroups were significantly lower when compared separately to controls or when compared to each other (Table 6). The severe group had the minimum values. The *E*' velocities and *A*' velocities were lower, and *E*'/*A*' and IVRT were greater significantly among moderate and severe asthmatic subgroups versus the control group. The previous diastolic indices were significantly different in the severe subgroup than the moderate subgroup or mild subgroup (Table 6).

**Table 5:** Comparison of the RV function of asthmatic children and controls by conventional echocardiography and TDE

Right ventricular function	Group I “Control” (*n* = 35)		Group II “Asthma” (*n* = 35)		*p*
	Min.–Max	Mean ± SD	Min.–Max	Mean ± SD	
Echocardiography					
TAPSE (cm)	1.48–2.71	2.10 ± 0.36	1.49–2.63	2.05 ± 0.29	0.580
*E* velocity (cm/sec)	49.0–82.0	60.46 ± 6.78	47.0–68.0	58.26 ± 4.92	0.125
*A* velocity (cm/sec)	36.0–51.0	44.26 ± 4.19	33.0–58.0	43.91 ± 5.05	0.758
*E*/*A* ratio	0.20–1.70	1.33 ± 0.23	1.12–1.77	1.33 ± 0.14	0.975
Pulsed-wave Tissue Doppler					
*Lateral* *leaflets*					
*S*' velocity(cm/sec)	13.70–17.40	15.71 ± 0.98	6.90–16.80	11.53 ± 3.24	< 0.001*
*E*' velocity (cm/sec)	6.40–17.40	16.02 ± 1.75	7.40–16.0	11.56 ± 3.18	< 0.001*
*A*' velocity(cm/sec)	0.70–11.60	10.42 ± 1.73	3.90–11.0	7.01 ± 2.53	< 0.001*
*E*'/*A*' ratio	1.30–1.59	1.49 ± 0.06	1.39–2.0	1.70 ± 0.18	< 0.001*
IVRT msec	92.20–119.0	102.39 ± 5.37	87.90–176.10	140.10 ± 34.35	< 0.001*

*r* Pearson coefficient

*Statistically significant at *p* ≤ 0.05

**Table 6:** Comparison between the asthmatic subgroups and control group according to TDE variables at lateral tricuspid annulus

Tissue Doppler data (RV)	Mild (*n* = 10)	Moderate (*n* = 16)	Severe (*n* = 9)	Control (*n* = 35)	*F*	*p*
S′ velocity (cm/sec)						
Min.–Max	10.2–15.10	8.70–16.80	6.90–9.80	13.7–17.4	43.820*	< 0.001*
Mean ± SD	12.63 ± 1.87	12.87 ± 3.30	7.94 ± 0.89	15.74 ± 0.98		
Median	13.15	14.15	8.10	15.80		
*p*_1_	< 0.001*	< 0.001*	< 0.001*			
Sig.bet.Grps	*p*_2_ = 0.989, *p*_3_ < 0.001*, *p*_4_ < 0.001*					
*E′* velocity (cm/sec)						
Min.–Max	12.50–16.0	8.20–15.90	7.40–9.30	6.40–17.40	44.385*	< 0.001*
Mean ± SD	14.53 ± 1.51	11.54 ± 3.01	8.30 ± 0.62	16.02 ± 1.75		
Median	15.15	10.55	8.30	16.20		
*p*_1_	0.171	< 0.001*	< 0.001*			
Sig.bet.Grps	*p*_2_ = 0.002*, *p*_3_ < 0.001*, *p*_4_ = 0.001*					
A′ velocity (cm/sec)						
Min.–Max	7.50–11.0	4.90–11.0	3.90–4.80	0.70–11.60	35.823*	< 0.001*
Mean ± SD	9.28 ± 1.48	7.13 ± 2.26	4.26 ± 0.32	10.42 ± 1.73		
Median	9.24	6.10	4.30	10.60		
*p*_1_	0.268	< 0.001*	< 0.001*			
Sig.bet.Grps	*p*_2_ = 0.016*, *p*_3_ < 0.001*, *p*_4_ = 0.001*					
*E*/′*A′* ratio						
Min.–Max	1.39–1.89	1.40–1.83	1.81–2.0	1.30–1.59	52.224*	< 0.001*
Mean ± SD	1.57 ± 0.14	1.64 ± 0.13	1.93 ± 0.08	1.49 ± 0.06		
Median	1.56	1.66	1.97	1.50		
*p*_1_	0.073	< 0.001*	< 0.001*			
Sig.bet.Grps	*p*_2_ = 0.295, *p*_3_ < 0.001*, *p*_4_ < 0.001*					
IVRT msec						
Min.–Max	87.0–90.0	95.0–170.2	194.2–176.1	94.2–119.0	83.861*	< 0.001*
Mean ± SD	105.0 ± 9.85	148.3 ± 28.7	171.8 ± 3.7	102.39 ± 5.37		
Median	150.61	160.70	169.90	102.0		
*p*_1_	0.876	< 0.001*	< 0.001*			
Sig.bet.Grps	*p*_2_ < 0.001*, *p*_3_ < 0.001*, *p*_4_ = 0.001*					

*F*: *F* for ANOVA test; pairwise comparison bet. each of the 2 groups was done using post hoc test (Tukey)

*p*: *p* value for comparing between the studied groups

*p*_1_: *p* value for comparing between control and each other group

*p*_2_: *p* value for comparing between mild and moderate

*p*_3_: *p* value for comparing between mild and severe

*p*_4_: *p* value for comparing between moderate and severe

*Statistically significant at *p* ≤ 0.05

**Fig. 2:**
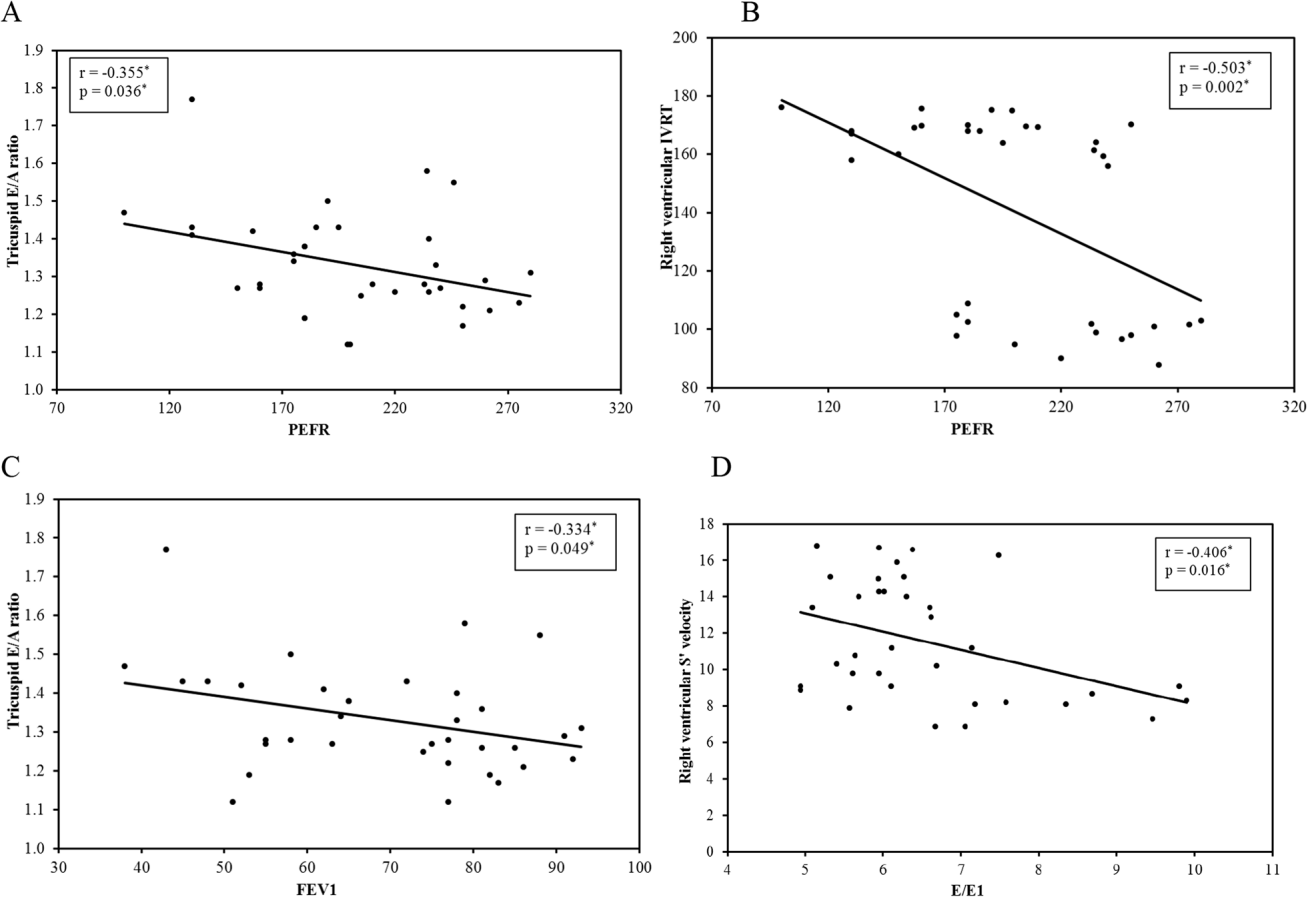
Correlation of lateral tricuspid annulus indices with risk factors among asthmatic group. **A** Correlation of PEFR with *E*'/*A*' at lateral tricuspid annulus among asthmatic group. **B** Correlation of PEFR with IVRT at lateral tricuspid annulus among asthmatic group. **C** Correlation of FEV1 with lateral *E*'/*A*' at tricuspid annulus among asthmatic group. **D** Correlation of *E*/*E*' of medial mitral annulus with at *S*' velocity at lateral tricuspid annulus among asthmatic group

Figure 2–-D shows the negative correlation between pulmonary function test (PEFR) and RV diastolic variable measures of asthmatic group (IVRT and *E*'/*A*'), respectively (*P* = 0.002 and *r* = −0.503*) (*P* = 0.036 and r =−0.355*). It also clarified the negative correlation between RV function (*S*' and *E*') and LV filling pressure (*E*/*E*').

Here, about 90% of severe asthmatics had impaired RV function (systolic, diastolic, or both). They had lower values than the recommended cutoff values for children at either S' wave velocity, *E*', and/or *A*' velocity at the tricuspid annulus [13]. Meanwhile, 31.2% of moderate asthmatics had lower *S*' values (systolic), and 56.2% had abnormal *E*’ and *A*’ values (diastolic). Although the IVRT of the mild group was significantly greater than the controls, none of the mild group showed abnormal LV or RV function. In contrast, the LV function indices were still normal for all asthmatic groups.

## Discussion

Bronchial asthma is a significant global health problem with rising prevalence especially in developing countries. It increases the cost of care and raises the burden on patients and society [2, 14]. Asthma affects the lung and other organs including the heart. Right ventricular dysfunction was found in a considerable percentage of asthmatic children and was attributed to pulmonary hypertension [4, 5]. The impact of asthma as a chronic disease on the biventricular cardiac performance is still challenging.

Cases and controls were properly matched in terms of age, height, and weight with no statistically significant differences in respiratory rate and/or heart rate. This is consistent to the study done by Shedeed et al. and Mahmoud et al. [8, 15]. Pulmonary function tests (PEFR and FEV1) in asthmatic children showed significantly lower values than controls. Similar to Ghaderian et al., the values of FEV1 and PEFR were significantly lower in the severe asthmatic subgroup than controls and/or to the mild asthmatics subgroup [16]. In agreement with Shedeed et al., conventional echocardiography variables of LV and RV function (EF, FS, TAPSE, peak *E* wave velocity, peak *A* velocity, and *E*/*A* ratio) were insignificantly different among asthmatic patients and control cases (*P* > 0.05) [8]. On the contrary, Abdalla and El Azeem studies had reported impired LV diastolic function in children with bronchial asthma by conventional echocardiography despite absence of impired RV function [17]. The conventional echocardiography in our wrok demonsterated insignificant difference regarding the echocardiographic variables of both ventricles. However, TDE revealed a significant difference in many parameters (systolic and/or diastolic) between asthmatic and control groups.

The LV function was preserved for all cases. Studies of TDE revealed that the medial mitral annulus indices of asthmatics (*S*' velocity and peak *E*)' were significantly reduced, while the IVRT and *E*/*E*' were significantly increased in asthmatics versus controls. The only significant change of mitral lateral annulus indices was in prolongation of IVRT among asthmatic. This was consistent with many studies such as Mohammed et al. at 2021 and Zeybek et al. in 2007 [7, 18]. However, Mohammed et al. reported fewer indices (peak *A*’ velocity) of both septal and lateral mitral leaflets in the asthmatic group [18].

We found a positive correlation between *E*' and *S*' medial mitral annulus with PERF and FEV1, respectively. A negative correlation was found between the *E*/*E*1 ratio (left ventricular filling pressure) and the PEFR and FEV1 (*P* > 0.5). This supports the indirect effect of poor pulmonary function tests in asthmatic children linked to the degree of severity of asthma on impairing the preload of the heart and hence the diastolic and/or systolic dysfunction.

The TDE study of RV revealed significant reductions in the S' velocity peak E' and the peak *A*' velocity of tricuspid lateral annulus of asthmatic group versus controls. There was a significant increase in IVRT and E'/A' versus the control group (*p* value < 0.05). These findings result in subclinical RV dysfunction among 74% of the asthmatic group. These data highlight the role of TDE in early detection of ventricular impairment. Our results are consistent with many studies [8, 18, 19].

El Masry et al. suggested that asthmatic children may experience bouts of transient pulmonary hypertension during exacerbations of respiratory symptoms, which may exert a cumulative effect leading to RV hypertrophy and may explain subclinical RV dysfunction [20]. Thus, proper control of asthmatic children was recommended.

Our study showed a significant negative correlation between PEFR (*P* > 0.5) and the *E*'/*A*' ratio and IVRT, respectively, for the tricuspid lateral annulus, which reflects the impact of poor pulmonary function tests on diastolic function of RV. These data agree with by Ozdemir et al. who reported the same correlation in asthmatic children [21].

Interestingly, we found a significant negative correlation between the (*E*/*E*1) of LV and the (*S*'‐wave, *E*'‐wave, *A*'‐wave) of RV. This explains the RV to LV link, referring to the effect of impaired systolic and/or diastolic function of RV on impairing the left ventricular filling pressure, and thus the LV preload [18].

We also studied the myocardial performance in mild, moderate, and severe asthma. Severe asthmatic cases represented 25.7% of asthmatics, and ~ 90% of severe asthmatics showed impaired subclinical RV function not detected by conventional echocardiography with normal LV function. The main TDE variables involved in the lateral tricuspid were E'‐wave and *A*’‐wave. The *S*'‐wave was significantly lower, and the *E*'/*A*' and IVRT were significantly higher than controls similar to other studies [7, 18, 22].

The moderate asthmatic patients represented 45.5% of cases. About 56% of them had impaired diastolic function, and 33% had impaired systolic function of RV. The moderate subgroup showed significantly different TDE variables for the lateral tricuspid (*S*'‐wave, *E*'‐wave, *A*'‐wave, the *E*'/*A*', and IVRT). These results are similar to other studies reporting early RV dysfunction in severe and or moderate asthmatics [7, 9, 18]. The TDE indices of RV function of severe subgroups were significantly reduced versus mild or moderate subgroups. In addition, significantly prolonged IVRT of medial and lateral mitral leaflets (LV) was found among severe and moderate asthmatics versus controls.

Here, the IVRT measured by TDE played an essential role for evaluation in many aspects. It is correlated with pulmonary function and is a tool for comparison between asthma subgroups. The IVRT is also an early marker of impaired cardiac performance. Our data for the mild asthmatic subgroup were surprising: A significant prolongation of IVRT was seen for both RV and LV versus controls with preserved biventricular function. This reflects the multiplicity of contributing factors underlying impaired cardiac diastolic or systolic function among asthmatics. The severity of chronic asthma is not the only risk for cardiac dysfunction—even mild asthmatics are not safe. We noted impairments in mild patients might be attributed to previously poor controlled severe exacerbations. These impairments might affect the cardiac function during severe exacerbation and remain hidden until our study. Our results were similar to De-Paula et al. and Ozde [23, 24]. In addition, RV diastolic dysfunction in mild-to-moderate cases was documented by speckle tracking echocardiography as reported by Abdel Mohsen et al. [25]. Further studies are needed to confirm and explain these findings and to evaluate the clinical implications of these abnormalities.

## Conclusions

Cardiac dysfunction was detected by Tissue Doppler Echocardiography rather than conventional echocardiography in chronic asthmatic children. Right ventricular dysfunction was earlier and more aggressive than LV dysfunction and directly related to poor pulmonary function tests. Therefore, TDE is the recommended imaging modality in asthmatic patients regardless of the degree of asthma severity. These cases should be screened by IVRT measured by TDE together with regular PEFR for early detection of cardiac and pulmonary dysfunction because these metrics are significantly and negatively correlated with each other and also easy to perform.

## Data Availability

The datasets used and analyzed during the current study are available from the corresponding author on reasonable request.

## Abbreviations

RV: Right ventricle
LV: Left ventricle
PH: Pulmonary hypertension
TDE: Tissue Doppler echocardiography
PEFR: Pulmonary expiratory flow rate
FEV1: Forced expiratory volume in first second
EF: Ejection fraction
FS: Fractional shortening
TAPSE: Tricuspid annular plane systolic excursion
IVRT: Isovolumic relaxation time
